# Prognostic value of multivariate logistic regression analysis and amyloid A lactate monitoring in patients with severe pneumonia-associated sepsis

**DOI:** 10.1186/s12890-025-03648-3

**Published:** 2025-04-23

**Authors:** Mengying Xie, Zuliang Min, Wei Jiang, Zhifang He, Xuexia Xia

**Affiliations:** 1 Emergency Department, Wuxi Ninth People’s Hospital Affiliated to Soochow University, 999 Liangxi Road, Binhu District, Wuxi, 214062 Jiangsu Province China; 2 Pediatric Department, Wuxi Ninth People’s Hospital Affiliated to Soochow University, Wuxi, 214062 Jiangsu Province China

**Keywords:** Severe pneumonia, Sepsis, Amyloid A, Lactate, Prognosis

## Abstract

**Background:**

Patients with severe pneumonia-associated sepsis often face high mortality rates, highlighting the need for simple and effective prognostic biomarkers. This study aimed to investigate the prognostic significance of serum amyloid A (SAA) and blood lactate (Lac) levels using multivariate logistic regression.

**Method:**

This was a retrospective study conducted from January 2021 to December 2023, which included 156 patients diagnosed with severe pneumonia. Of these, 54 developed sepsis (septic group) while 102 did not (non-septic group). Clinical data, SAA, and Lac levels were compared between the groups. Multivariate logistic regression was employed to identify factors influencing the onset of severe pneumonia-associated sepsis and to assess the prognostic significance of SAA and Lac.

**Result:**

Significant differences were found in APACHE II score, SOFA score, age, mechanical ventilation, SAA, and Lac levels between the septic and non-septic groups (*P* < 0.05). Logistic regression analysis identified age, SOFA score, APACHE II score, mechanical ventilation, SAA, and Lac as influencing factors for severe pneumonia-associated sepsis (*P* < 0.05). Patients with poor prognosis (PP) had significantly elevated SAA and Lac levels compared to those with good prognosis (GP) (*P* < 0.05). Among septic patients, those with PP had significantly higher SAA and Lac levels compared to those with GP (*P* < 0.05). Multivariate logistic regression revealed that advanced age, septic shock, elevated SAA levels, and increased Lac levels were predictors of PP (*P* < 0.05). The prognostic value of SAA and Lac was demonstrated by AUCs of 0.764 and 0.771, respectively. When combined, the AUC increased to 0.903 with a specificity of 95.00% and sensitivity of 80.25%.

**Conclusion:**

Severe pneumonia-associated sepsis is influenced by age, SOFA score, APACHE II score, mechanical ventilation, SAA, and Lac levels. Elevated SAA and Lac levels are associated with PP and can provide prognostic information for adverse outcomes. While SAA and Lac show potential as biomarkers for predicting the prognosis of severe pneumonia-associated sepsis, their clinical utility should be considered in conjunction with other diagnostic and clinical factors for effective patient management and risk stratification.

## Introduction

Pneumonia, caused by bacterial, viral, or fungal infections, leads to alveolar inflammation and respiratory impairment. The mortality rate of pneumonia accounts for about 75% of the mortality rate of acute respiratory infection [1]. Severe pneumonia is caused by the progression of pulmonary inflammation and malignant aggravation. As the disease progresses, patients may experience numerous intrapulmonary and extrapulmonary complications, among which sepsis is a prevalent extrapulmonary complication in severe pneumonia. Once sepsis sets in, it can readily lead to multiple organ dysfunction, failure, and shock, posing a grave threat to patients’ lives and safety [2, 3]. There is a widely held belief that the optimal window for treatment following sepsis onset is within the first hour. Initiating fluid resuscitation and anti-infective cluster therapy during this critical period has been deemed effective [4]. Hence, it is crucial to promptly and accurately assess and identify the onset of sepsis and implement effective preventive measures. While severe pneumonia infection is acknowledged as a leading cause of sepsis, there remains uncertainty regarding other contributing factors to sepsis [5]. Therefore, exploring the risk factors of severe pneumonia-related sepsis is really considerable to improve the prognosis of individuals with severe pneumonia-related sepsis. While numerous studies have been conducted in the past, the variations in trial design and observation indicators among previous studies have prevented the drawing of convincing and consistent conclusions. Based on this, it is still necessary to continue to carry out relevant research.

Clinical reports show a 19.0% higher mortality rate in patients with severe pneumonia-associated sepsis compared to those with severe pneumonia alone [6]. Currently, various critical scoring systems and imaging examination methods are employed for identifying critical illness and forecasting poor prognosis (PP). However, due to the extensive itemization in scoring systems, swift evaluation becomes challenging, and imaging techniques are constrained by economic and logistical limitations. Consequently, their widespread adoption in grassroots hospitals, particularly in resource-constrained regions, remains unattainable. In recent years, there has been a surge of interest in biomarkers characterized by their ease of acquisition and rapid detection, presenting a promising clinical avenue for enhanced prognostication. Serum amyloid A (SAA) is an apolipoprotein primarily synthesized by the liver, with limited production occurring elsewhere in the body. Its expression significantly increases in response to inflammatory stimuli such as infection, trauma, or inflammation. SAA serves as a novel marker indicative of early inflammation in infectious diseases [7, 8]. Blood lactic acid level (Lac) is the intermediate product of glucose metabolism, and the abnormal increase can reflect the degree of poor tissue perfusion and insufficient oxygen supply in sepsis [9]. SAA and Lac are elevated in sepsis and serve as useful predictive biomarkers [10]. Indeed, the prognostic significance of severe pneumonia-associated sepsis remains in the exploratory phase, underscoring the need for further in-depth research in this area. Therefore, this study initially examined the risk factors associated with severe pneumonia-related sepsis using logistic regression analysis. Subsequently, it monitored the levels of Lac and SAA in individuals with poor prognoses due to severe pneumonia-related sepsis and assessed their prognostic significance. The aim is to offer early detection of severe pneumonia-related sepsis and inform strategies for enhancing patient prognosis and reducing mortality rates.

## Subjects and methods

### Subjects

This retrospective study involved patients treated at our hospital from January 2021 to December 2023 who had severe pneumonia-associated sepsis. Data sources included electronic medical records, laboratory results, and radiologic findings.

#### Inclusion criteria

1. All the selected cases met the diagnostic criteria of severe pneumonia established by American Society of Infectious Diseases (Infectious Disease Society of America, IDSA) and American Thoracic Society (ATS). Diagnosis is confirmed if the primary criterion is met, along with at least one secondary criterion [11]. Primary Criterion: Mechanical ventilation is necessitated following intubation. Furthermore, vasoactive medications remain essential even after symptomatic management of septic shock. Secondary criteria: a). Respiratory rate ≥ 30 beats / min; b). Partial pressure of oxygen in arterial blood/concentration of oxygen in inhaled gas ≤ 250 mm /Hg; c). Multiple lobar infiltration; d). Disturbance of consciousness or orientation; e). Blood urea nitrogen (BUN) ≥ 7 mmol hand Lterf. Hypotension should be addressed with fluid resuscitation.
2. Age of patients ≥ 18 years;
3. Acute physiology and chronic health score (APACHE II) [12] > 11.

#### Exclusion criteria

1. Patients with other pulmonary diseases, such as pulmonary embolism and pulmonary tuberculosis;
2. Patients with other malignant tumors, such as non-small cell lung cancer;
3. Patients who expired within 24 h of admission;
4. Patients with severe immunosuppression or immunodeficiency;
5. Patients with infectious diseases other than pulmonary infections;
6. Patients with hematological diseases;
7. Patients who have been administered immunosuppressive drugs or antiplatelet drugs within the past month;
8. Patients with mental illness.

The study was approved by the institutional Review board at Wuxi Ninth People’s Hospital Affiliated to Soochow University (approval number WNH202402063).

### Grouping standard

Sepsis grouping criteria: infection combined sequential organ failure score (SOFA) ≥ 2, as defined in “International consensus on the definition of sepsis and septic shock (Sepsis-3.0)” [13]. In this study, among the 156 patients with severe pneumonia, 54 patients with sepsis (observed during hospitalization) were separated into sepsis group, and 102 patients without sepsis were assigned to the non-sepsis group.

Prognosis grouping criteria: The medical records of 54 individuals identified as having severe pneumonia-associated sepsis were retrieved from our hospital’s electronic medical record system. Subsequently, the individuals were categorized into two groupings depending on whether they deceased during hospitalization.34 of the 54 patients died and 20 survived. They were split up into two groups, including GP group and PP group.

### Clinical information

Clinical information included age, body mass index (BMI), gender, history of drinking and smoking, presence of diabetes, heart disease, and hypertension, respiratory rate, mean arterial pressure, Acute Physiology and Chronic Health Evaluation II (APACHE II) score, oxygenation index, white blood cell count, levels of procalcitonin (PCT) and C-reactive protein (CRP), blood culture results, and need for mechanical ventilation. 24 h after admission, all baseline data and clinical indicators were gathered and examined. Detection of serum SAA and Lac: Within 24 h of admission, 2 mL of fasting elbow venous blood was collected from each patient, and serum was obtained following centrifugation (3500 rpm, centrifugation radius 8 cm, for 15 min). The lactate (Lac) level was determined using an enzyme-linked immunosorbent assay (ELISA) (Lactate Assay Kit, SolarBio, China), while SAA level was measured using turbidimetry (WZB-172 Turbidimeter, Leici, China). All procedures followed the provided instructions to ensure accuracy and reproducibility. The normal range for Lac was 0.5 to 1.7 mmol/L, and for SAA, it was 0 to 10 mg/L, based on the manufacturer’s guidelines. The cutoff value for Lac was determined to be 2.0 mmol/L, and for SAA, it was set at 10 mg/L, as detailed in the statistical analysis section.

SAA testing was included in this study based on its superior clinical utility in early sepsis detection compared to standard biomarkers like CRP and PCT, as shown in previous studies. Although CRP and PCT are routinely used, SAA was chosen due to its ability to provide more timely diagnostic insights in sepsis, especially in its early stages. The cost-effectiveness of SAA testing was comparable to that of PCT, with potential advantages in certain clinical settings. SAA testing was performed selectively as part of the study protocol and was not a routine hospital test. The results of SAA testing significantly influenced clinical decision-making, particularly in patients with ambiguous sepsis diagnoses, guiding the early initiation of sepsis-specific therapies, including antibiotic treatment and fluid resuscitation.

Additionally, information regarding corticosteroid treatment, criteria for antibiotic therapy, and the use of non-invasive ventilation was meticulously recorded.

Corticosteroid treatment: Patients received corticosteroids based on clinical indications such as severe inflammation or septic shock [14, 15].

Criteria for antibiotic therapy: Administered according to the guidelines for severe pneumonia, focusing on the pathogen identified or suspected and local resistance patterns.

Use of non-invasive ventilation: Applied in patients deemed at risk of respiratory failure but not immediately requiring invasive mechanical ventilation.

### Observation index

1. Comparison of the clinical data and serum levels of SAA and Lac between sepsis group and non-sepsis group;
2. To retrospectively analyze the factors affecting the occurrence of severe pneumonia-associated sepsis through multivariate logistic regression;
3. Comparison of clinical data between patients with favorable and unfavorable prognosis, with collection of serum SAA and Lac levels from existing data;
4. Multivariate logistic regression was used to analyze the variables that contributed to the PP of severe pneumonia-associated sepsis in retrospect.
5. To evaluate the predictive usefulness of serum SAA and Lac levels for assessing the PP of severe pneumonia-associated sepsis, a Receiver Operating Characteristic (ROC) curve analysis was conducted.

### Research flow chart

**Fig. 1:**
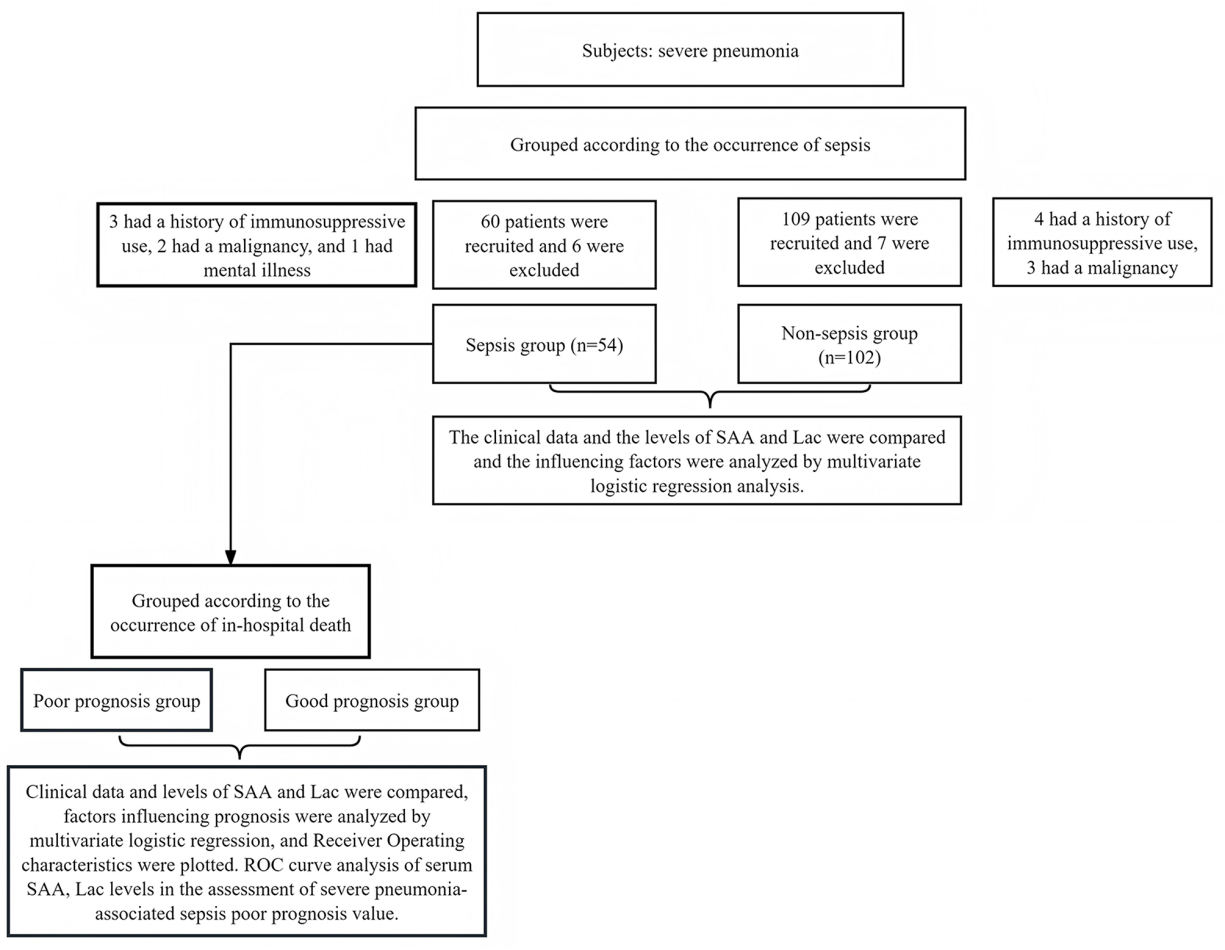
Flow chart of this study

The Flow chart of this study is seen in Fig. 1.

### Statistical methods

The data were analyzed by SPSS22.0 statistical software, and the pictures were processed by GraphPadPrism9. The measurement data were represented by ($\bar x \pm s$) with a uniform variance and a normal distribution, and the independent sample t-test was used to compare the groups. The χ2 test was used to test the counting data, which were reported as [n (%)]. Logistic regression was conducted using sepsis occurrence as the dependent variable (1 = sepsis, 0 = no sepsis) and significant univariate predictors (*P* < 0.05) as independent variables. SPSS 22.0 was used for data analysis with a significance threshold at *P* < 0.05.

## Results

### Univariate analysis

Significant differences were observed between the septic and non-septic groups in terms of age, SOFA score, APACHE II score, mechanical ventilation, SAA, Lac levels, ARDS status, and P/F ratio (*P* < 0.05, Table 1), while other parameters, including body mass index, gender, smoking history, history of drinking, high blood pressure, diabetes, coronary artery disease, respiratory rate, mean arterial pressure, oxygenation index, body temperature, white blood cell count, CRP, PCT, and COVID-19 status, showed no significant differences (*P* > 0.05).

**Table 1:** Univariate analysis of patients with severe pneumonia-associated sepsis and patients without sepsis

Items	Sepsis group (*n* = 54)	Non-sepsis group (*n* = 102)	t/χ^2^	*P*
Age (years)	62.58 ± 5.92	54.36 ± 4.74	9.436	<0.001
Body mass index (kg/m^2^)	22.52 ± 3.19	22.47 ± 3.21	0.093	0.926
Gender			2.104	0.147
Male	22 (40.74)	54 (52.94)		
Female	32 (59.26)	48 (47.06)		
Smoking history			2.563	0.109
Yes	18 (33.33)	22 (21.57)		
No	36 (66.67)	80 (78.43)		
History of drinking			1.686	0.194
Yes	22 (40.74)	31 (30.39)		
No	32 (59.26)	71 (69.61)		
High blood pressure			2.563	0.109
Yes	18 (33.33)	22 (21.57)		
No	36 (66.67)	80 (78.43)		
Diabetes			0.422	0.516
Yes	31 (57.41)	64 (62.75)		
No	23 (42.59)	38 (37.25)		
Coronary artery disease			0.008	0.928
Yes	29 (53.70)	54 (52.94)		
No	25 (46.30)	48 (47.06)		
Respiratory rate (times)/min)	33.57 ± 5.87	32.33 ± 5.92	1.248	0.214
Mean arterial pressure (mmHg)	82.52 ± 12.47	82.47 ± 12.52	0.024	0.981
SOFA Scoring	5.74 ± 0.85	3.63 ± 0.37	21.552	<0.001
APACHE II Scoring	22.39 ± 4.74	14.55 ± 2.95	12.707	<0.001
Oxygenation index	154.52 ± 32.25	156.39 ± 32.47	0.343	0.732
Body temperature (℃)	37.90 ± 0.84	38.00 ± 0.87	0.691	0.491
White blood cell count (×10^9^/L)	20.52 ± 4.57	20.39 ± 4.52	0.170	0.865
CRP (mg/L)	76.58 ± 12.66	75.87 ± 12.59	0.334	0.738
PCT (ng/mL)	4.52 ± 0.57	4.64 ± 0.62	0.096	0.924
Etiological results			0.252	0.616
Positive	41 (75.93)	81 (79.41)		
Negative	13 (24.07)	21 (20.59)		
Mechanical ventilation			38.044	<0.001
Invasive	39 (72.22)	22 (21.57)		
Non-invasive	15 (27.78)	80 (78.43)		
SAA (mg/L)	29.52 ± 4.47	22.63 ± 3.81	10.111	<0.001
Lac (mmol/L)	8.24 ± 2.17	6.12 ± 1.59	6.956	<0.001
COVID-19 Status	Positive: 30 (55.56%)	Positive: 40 (39.22%)	1.633	0.201
ARDS Status	Present: 35 (64.81%)	Present: 22 (21.57%)	19.647	< 0.001
P/F Ratio	180.65 ± 40.58	210.50 ± 39.84	3.876	< 0.001

### Multivariate analysis

Taking the occurrence of severe pneumonia-related sepsis as the variable that is dependent (occurrence = 1, non-occurrence = 0) and the index of single factor (*P* < 0.05) as the independent variable (Table 2 for the assignment table). Multivariate logistic regression conducted using sepsis occurrence as the dependent variable (1 = sepsis, 0 = no sepsis) identified age, SOFA score, APACHE II score, invasive mechanical ventilation, SAA, and Lac as significant predictors of severe pneumonia-related sepsis (*P* < 0.05, Table 3).

**Table 2:** Assignment table

Items	Variable type	Assignment
Age	Continuity variable	Original value input
SOFA Scoring	Continuity variable	Original value input
APACHE II Scoring	Continuity variable	Original value input
Mechanical ventilation	Binomial variable	Invasive = 1, Non-invasive = 0
SAA	Continuity variable	Original value input
Lac	Continuity variable	Original value input

**Table 3:** Logistic regression analysis of the occurrence of severe pneumonia-associated sepsis

Factors	Regression coefficient	Standard error	Waldχ^2^ Value	*P*	OR	95%CI	
						Lower limit	Upper limit
Age	1.574	0.547	8.280	0.004	4.826	1.652	14.099
Elevated SOFA score	1.691	0.618	7.487	0.006	5.425	1.616	18.216
Increased APACHE II score	1.125	0.367	9.397	0.002	3.080	1.500	6.324
Requirement for invasive mechanical ventilation	1.364	0.521	6.854	0.009	3.912	1.409	10.861
Elevate SAA	1.252	0.411	9.280	0.002	3.497	1.563	7.827
Elevate Lac	0.847	0.264	10.293	0.001	2.333	1.390	3.914

### Comparison of clinical data between PP group and GP group in patients with severe pneumonia-associated sepsis

There were significant differences in age and septic shock between PP group and GP group (*P* < 0.05). There wasn’t a noticeable variation in coronary heart disease, diabetes, hypertension, drinking history, smoking history, sex, body mass index, respiratory rate, mean arterial pressure, SOFA score, APACHEII score, oxygenation index, body temperature, white blood cell count, CRP, PCT, pathogenic results and mechanical ventilation involving the two groupings (*P* > 0.05), as seen in Table 4.

**Table 4:** Comparison of clinical data between PP group and GP group in patients with severe pneumonia-associated sepsis

Items	Poor prognosis group (*n* = 34)	Group with good prognosis (*n* = 20)	t/χ^2^	*P*
Age (years)	69.49 ± 6.37	56.36 ± 4.74	7.995	<0.001
Body mass index (kg/m^2^)	22.47 ± 3.22	22.45 ± 3.19	0.022	0.982
Gender			0.434	0.510
Male	15 (64.81)	7 (35.00)		
Female	19 (35.19)	13 (65.00)		
Smoking history			0.159	0.690
Yes	12 (22.22)	6 (30.00)		
No	22 (77.78)	14 (70.00)		
History of drinking			0.434	0.510
Yes	15 (44.12)	7 (35.00)		
No	19 (55.88)	13 (65.00)		
High blood pressure			0.159	0.690
Yes	12 (35.29)	6 (30.00)		
No	22 (64.71)	14 (70.00)		
Diabetes			0.749	0.387
Yes	18 (52.94)	13 (65.00)		
No	16 (47.06)	7 (35.00)		
Coronary artery disease			3.393	0.065
Yes	15 (44.12)	14 (70.00)		
No	19 (55.88)	6 (30.00)		
Respiratory rate (times/min)	33.48 ± 5.79	33.14 ± 5.75	0.209	0.835
Mean arterial pressure (mmHg)	81.36 ± 12.31	81.74 ± 12.35	0.109	0.913
SOFA Scoring	5.69 ± 0.87	5.72 ± 0.92	0.119	0.905
APACHE II Scoring	22.47 ± 4.81	22.52 ± 4.85	0.037	0.971
Oxygenation index	155.39 ± 32.74	155.87 ± 32.87	0.052	0.959
Body temperature (℃)	37.52 ± 0.87	37.57 ± 0.89	0.202	0.841
White blood cell count (×10^9^/L)	21.57 ± 4.63	21.47 ± 4.67	0.076	0.939
CRP (mg/L)	76.42 ± 12.57	75.91 ± 12.63	0.144	0.886
PCT (ng/mL)	4.49 ± 0.59	4.81 ± 0.62	1.889	0.064
Etiological results			2.075	0.150
Positive	28 (75.93)	13 (65.00)		
Negative	6 (24.07)	7 (35.00)		
Mechanical ventilation			2.585	0.108
Invasive	22 (64.71)	17 (85.00)		
Non-invasive	12 (35.29)	3 (15.00)		
Septic shock			24.816	<0.001
Yes	31 (91.18)	5 (25.00)		
No	3 (8.82)	15 (75.00)		

### Comparison of serum SAA and Lac levels between PP group and GP group in individuals with severe pneumonia-associated sepsis

The levels of SAA and Lac in PP group were considerably higher than those in GP group (*P* < 0.05), as shown in Table 5.

**Table 5:** Comparison of serum SAA and Lac levels between PP group and GP group in individuals with severe pneumonia-associated sepsis

Group	SAA (mg/L)	Lac (mmol/L)
Poor prognosis group (*n* = 34)	32.49 ± 5.77	8.97 ± 2.31
Group with good prognosis (*n* = 20)	12.52 ± 3.21	5.12 ± 1.54
*t*	14.204	6.625
*P*	<0.001	<0.001

### Analysis of influencing factors of PP in individuals with severe pneumonia-associated sepsis

Using severe pneumonia-related sepsis occurrence as the dependent variable (1 for occurrence, 0 for non-occurrence), and the variables from Table 4 (*P* < 0.05) as independent variables (original input for age, binary for septic shock (1 for yes, 0 for no), original input for SAA, original input for Lac), multivariate logistic regression analysis revealed that age, septic shock presence, elevated SAA levels, and increased Lac levels were significant influencing factors associated with the PP of severe pneumonia-related sepsis (*P* < 0.05), as seen in Table 6.

**Table 6:** Analysis of influencing factors of PP in patients with severe pneumonia-associated sepsis

Factors	Regression coefficient	Standard error	Wald^2^ value	*P*	OR	95%CI	
						lower limit	upper limit
Elevation in age	2.158	0.947	5.193	0.023	8.654	1.352	55.375
Presence of septic shock	1.257	0.369	11.604	0.001	3.515	1.705	7.244
Rise in SAA level	0.974	0.214	20.715	<0.001	2.649	1.741	4.029
Rise in Lac level	1.149	0.314	13.390	<0.001	3.155	1.705	5.838

### The prognostic value of serum SAA and Lac alone or in combination in patients with severe pneumonia-associated sepsis

**Fig. 2:**
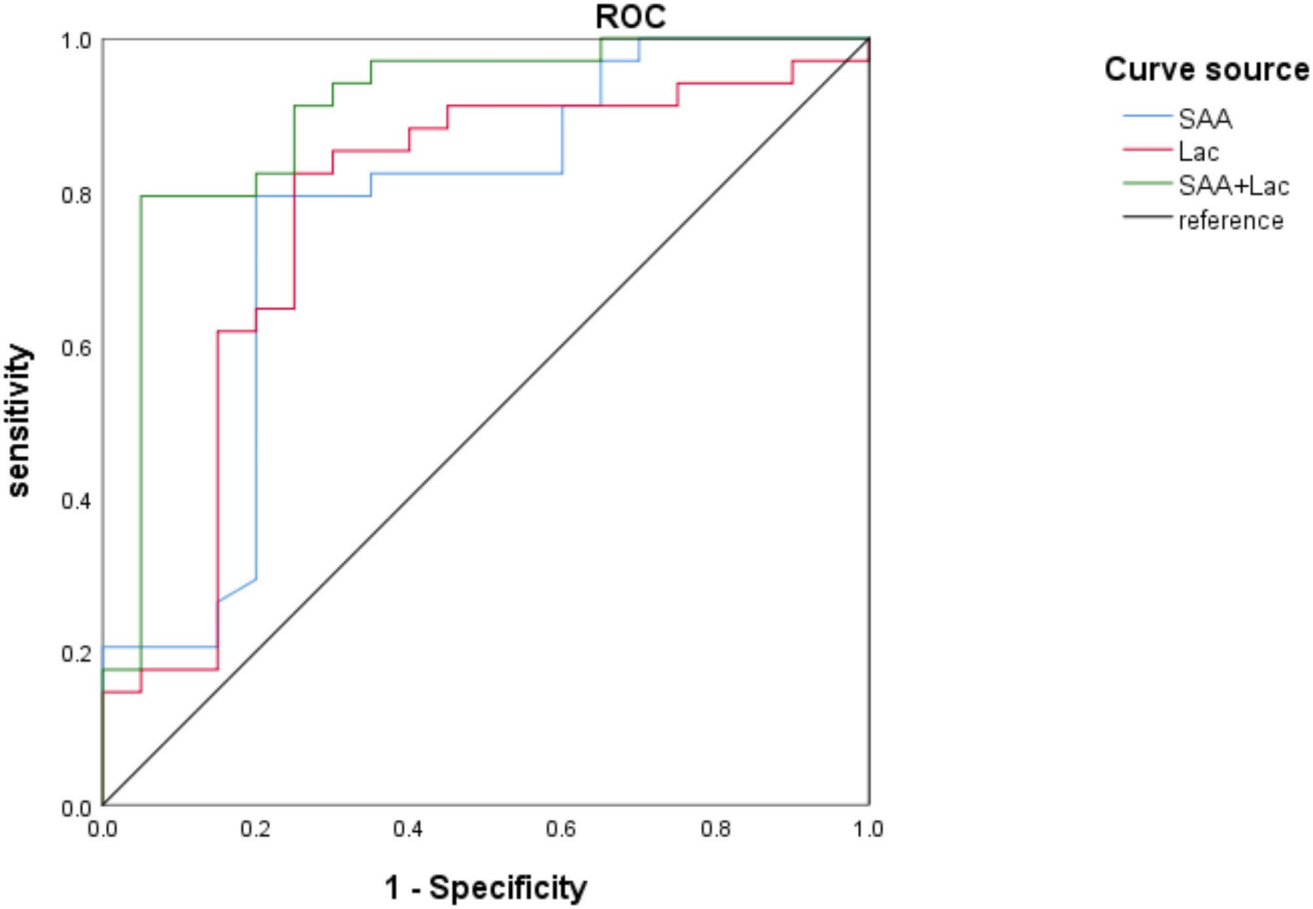
ROC curve of serum SAA and Lac alone or in combination to predict the prognostic value of individuals with severe pneumonia-associated sepsis

The AUC of SAA and Lac in evaluating the prognostic value of severe pneumonia-associated sepsis was 0.764 and 0.771, respectively. In addition, the highest AUC after pairwise combination was 0.903, with sensitivity and specificity of 80.25% and 95.00%, respectively, as shown in Table 7; Fig. 2.

**Table 7:** The value of serum SAA and Lac alone or in combination in predicting the prognosis of patients with severe pneumonia-associated sepsis

Factors	AUC	SE	95%CI	*P*	Truncated value	Youden index	Sensitivity	Specificity
SAA	0.764	0.073	0.622–0.906	0.001	18.880	0.594	79.40	80.00
Lac	0.771	0.072	0.629–0.913	0.001	6.655	0.574	79.47	75.00
SAA + Lac	0.903	0.047	0.810–0.970	<0.001	/	0.626	80.25	95.00

## Discussion

Severe pneumonia stands as the primary causative factor for sepsis, with sepsis itself emerging as a frequent complication of severe pneumonia [16]. The pathogenesis of sepsis is complex, including inflammatory reaction, immune dysfunction, abnormal coagulation function and so on, involving pathophysiological changes of many systems and organs [17]. In response to severe infection, monocytes, pivotal in maintaining circulatory function, undergo abnormal expression patterns mediated by proinflammatory factors. This, in turn, impacts the activation of the systemic coagulation cascade [18, 19]. Additionally, the liver’s ability to produce coagulation factors is impaired, and fibrinolysis is obstructed by plasminogen activators. These disruptions, combined with endothelial dysfunction, lead to microcirculatory disturbances, organ failure, and shock [20, 21]. Thus, identifying sepsis etiology and its contributing factors is crucial for managing critically ill patients.

Logistic regression analysis showed that age, SOFA score, APACHEII score, invasive mechanical ventilation, SAA and Lac were the influencing factors of severe pneumonia-associated sepsis. As age advances, elderly individuals experience a decline in immune function, rendering vital organs more susceptible to damage and substantially elevating the risk of sepsis. Furthermore, the elderly often present with various chronic illnesses, further compounding their susceptibility to infections [22]. The SOFA score serves as a pivotal metric for assessing the onset of sepsis. Patients afflicted with severe pneumonia may incur lung injury subsequent to sepsis, thereby precipitating respiratory failure and exacerbating organ dysfunction. A higher SOFA score correlates with an augmented risk of organ dysfunction [23, 24]. SOFA score is a validated tool for diagnosing sepsis in critically ill patients [25]. APACHEII score is a clinical scoring system to evaluate patients’ acute physiological and chronic health status, which is accustomed to evaluate the severity and prognosis of severe individuals [26]. An elevated APACHE II score typically signifies heightened disease severity in patients, concomitant with more pronounced organ dysfunction and metabolic disturbances. In instances of sepsis, a high APACHE II score may indicate the gravity of the infection [27]. Mechanical ventilation serves as a standard adjunctive therapy for severe pneumonia patients; however, invasive mechanical ventilation disrupts the natural respiratory tract defense mechanisms. The placement of tracheal intubation poses a risk of bacterial colonization, potentially leading to respiratory tract infections, intubation-associated pneumonia, and ventilation-induced lung injuries. Additionally, invasive ventilation can escalate the body’s inflammatory response, exacerbate systemic inflammatory response syndrome, and heighten the likelihood of sepsis [28].

SAA levels rise early in response to inflammation and can serve as a timely diagnostic marker, although its diagnostic value is moderate compared to other inflammatory markers like CRP and PCT. Its role should be considered supplementary, in the clinical decision-making process [29]. SAA typically peaks 8 to 18 h after the onset of sepsis, with a sensitivity of approximately 76.4% for early diagnosis [30]. The findings of this study revealed that the levels of SAA were notably higher in the sepsis group compared to non-septic patients. This elevation could be attributed to the acute inflammatory or infectious state experienced by patients with severe pneumonia, leading to increased synthesis of SAA or metabolic pathway obstruction, resulting in a significant upregulation of SAA expression in septic patients. Lac, produced by anaerobic glucose metabolism, is elevated in sepsis due to impaired oxygen delivery and tissue metabolic demand, increasing Lac levels [31]. After sepsis, reduced tissue perfusion and liver dysfunction hinder Lac clearance, leading to accumulation in the blood and worsening the condition [32]. Hence, this study identified that the elevation of Lac levels serves as a contributing factor to the development of sepsis.

This study further analyzed the prognosis of severe pneumonia-associated sepsis. Age, septic shock, and elevated SAA and Lac levels were identified as risk factors for PP. Aging leads to a decline in organ function, making elderly patients more vulnerable to infections and sepsis, thereby increasing mortality risk. Furthermore, elderly patients often present with multiple comorbidities and complex medication regimens. Managing sepsis in this population may be complicated by challenges such as inappropriate drug selection and poor drug tolerance, thus exacerbating the treatment difficulty and increasing mortality rates. Septic shock, a severe manifestation of sepsis, poses a grave threat to patients’ lives. It precipitates a systemic inflammatory response that impairs the function of vital organs, including the heart, lungs, and kidneys, further deteriorating the patient’s condition and heightening the risk of mortality [9]. The analysis reveals elevated serum levels of SAA and Lac in sepsis patients. Prolonged elevation of SAA levels can induce amyloidosis, leading to deposition in internal organs and culminating in severe complications associated with chronic inflammatory conditions. Moreover, SAA is implicated in mediating various signaling pathways involved in the body’s inflammatory response, thereby exacerbating the progression of various infectious diseases. SAA serves as an endogenous ligand for Toll-like receptor 4/2, exerting inhibitory effects on pathway modification and contributing to immunomodulation [7]. The study findings indicate that SAA levels were higher in the PP group. The AUC for SAA in predicting the prognosis of severe pneumonia-associated sepsis was 0.764, with a sensitivity of 79.40% and specificity of 80.00%. These findings imply that SAA may influence the prognosis of severe pneumonia-associated sepsis and aid in prognostic evaluation of the disease.

Following the onset of severe pneumonia-associated sepsis, the body faces challenges in meeting metabolic demands due to compromised oxygen supply. As the pathophysiological cascade unfolds, the liver’s capacity to clear Lac diminishes significantly, resulting in Lac accumulation and subsequent acidosis. This impedes oxygen delivery, initiates a cascade of reactions, and hastens disease progression [33]. Septic survivors typically witness a normalization of Lac levels within 48 h post-onset. The study findings indicate that Lac levels are higher in the PP group, with an AUC of 0.771 for predicting the prognosis of severe pneumonia-associated sepsis. The sensitivity and specificity are 79.47% and 75.00%, respectively, suggesting that Lac might be involved in the occurrence of PP in severe pneumonia-associated sepsis. Furthermore, the combined AUC of SAA and Lac in predicting PP is 0.903, with a sensitivity of 80.25% and a specificity of 95.00%, surpassing the AUC when used alone. Active monitoring of SAA and Lac levels is recommended upon the onset of severe pneumonia-associated sepsis. However, it is important to emphasize that these biomarkers should be considered alongside other clinical assessments to inform treatment strategies, as relying solely on them may not provide sufficient predictive accuracy for individual patient outcomes.

In conclusion, severe pneumonia-associated sepsis is closely associated with aging, SOFA score, APACHE II score, invasive mechanical ventilation, SAA, and Lac levels. Elevated levels of SAA and Lac are associated with PP and provide valuable prognostic information for predicting adverse outcomes. However, it is important to note that while SAA and Lac can offer additional insights, their diagnostic and prognostic value should be considered alongside other clinical parameters. This study provides important perspectives on the early identification of patients at risk for sepsis and highlights the complexity of managing the progression from severe pneumonia to sepsis in clinical practice. Moreover, it emphasizes the need for a comprehensive approach to addressing the challenges related to the prognosis of severe pneumonia-associated sepsis. In addition, the limitations of this study include its single-center design and the relatively small study population, which may reduce the generalizability of the results. Further, while most patients had an etiological identification, a detailed breakdown of the infectious agents was not provided and could be explored in future studies. Due to the limited scope of monitoring SAA and Lac levels at a single time point, the study may present a unilateral perspective on prognosis. Future research should incorporate dynamic monitoring at multiple time points to provide a more comprehensive understanding of prognosis.

## Data Availability

The datasets used and/or analysed during the current study are available from the corresponding author on reasonable request.
